# Processing Irrelevant Location Information: Practice and Transfer Effects in a Simon Task

**DOI:** 10.1371/journal.pone.0064993

**Published:** 2013-07-04

**Authors:** Dan B. Welch, Aaron R. Seitz

**Affiliations:** 1 Department of Growth, Development and Structure, Southern Illinois University – School of Dental Medicine, Alton, Illinois, United States of America; 2 Department of Psychology, University of California Riverside, Riverside, California, United States of America; University of Melbourne, Australia

## Abstract

How humans produce cognitively driven fine motor movements is a question of fundamental importance in how we interact with the world around us. For example, we are exposed to a constant stream of information and we must select the information that is most relevant by which to guide our actions. In the present study, we employed a well-known behavioral assay called the Simon task to better understand how humans are able to learn to filter out irrelevant information. We trained subjects for four days with a visual stimulus presented, alternately, in central and lateral locations. Subjects responded with one hand moving a joystick in either the left or right direction. They were instructed to ignore the irrelevant location information and respond based on color (e.g. red to the right and green to the left). On the fifth day, an additional testing session was conducted where the task changed and the subjects had to respond by shape (e.g. triangle to the right and rectangle to the left). They were instructed to ignore the color and location, and respond based solely on the task relevant shape. We found that the magnitude of the Simon effect decreases with training, however it returns in the first few trials after a break. Furthermore, task-defined associations between response direction and color did not significantly affect the Simon effect based on shape, and no significant associative learning from the specific stimulus-response features was found for the centrally located stimuli. We discuss how these results are consistent with a model involving route suppression/gating of the irrelevant location information. Much of the learning seems to be driven by subjects learning to suppress irrelevant location information, however, this seems to be an active inhibition process that requires a few trials of experience to engage.

## Introduction

The sophisticated manner with which humans use their hands to manipulate objects is a result of a complex blend of sensory-motor control mechanisms. In particular, motor decisions require filtering sensory information to determine which information is relevant to the task at hand. This filtering task is made difficult because information that is relevant to some motor decisions can be irrelevant and even misleading, to other decisions. For example, responding by pressing a button on one’s left in response to a stimulus that appears in one’s right visual field requires suppressing a prepotent response to make a right-ward response. This is because in many other situations one will point or reach towards the location of observed objects. To examine this process, we studied learning in the context of the Simon task, a well-known behavioral assay of sensory-motor integration [1].

The Simon task was introduced by Simon and Small [2], who had subjects make left or right responses to low or high-pitched tones with key presses. The tones were presented either to the left or right ear. Responses to the "right" command (e.g., high-pitched tone) were 62 msec faster when it was presented in the right ear rather than the left ear; and the responses to the "left" command (e.g. low-pitched tone) were 60 msec faster when it was heard in the left ear rather than the right ear. The location of the auditory stimulus, although irrelevant to the task, directly influences response-selection. Simon and Small [2], argued that this is due to an automatic tendency to respond towards the source of the stimulation. The delay in reaction time that occurs when stimulus position and response position do not correspond is currently known as the Simon effect [3].

The Simon effect is much more than just an interesting observation. It is a behavioral assay that can be used to investigate perception, attention, action planning, and sensory-motor control mechanisms.

Within this context, is not surprising that the Simon effect has been found to exist for multiple sensory modalities. The Simon effect for visual stimuli has since been investigated and replicated several times using color [4]–[7]. It also has been obtained with a variety of other relevant stimulus dimensions [8] and geometric forms [9]. Taken together, the Simon effect seems to be a robust phenomenon that can be observed with a variety of stimuli.

Even though this effect is robust, it can be reduced through practice. This was first demonstrated in an early study conducted by Simon, Craft, and Webster [1]. They instructed subjects to press a left or a right key in response to a high or low pitched tone presented in the left or the right ear. The subjects performed 192 test trials a day for 5 days. They found that the magnitude of the Simon effect decreased from an average of 60 msec in the first session to 35 msec in the 5th session, but the effect was not eliminated. In a more recent study, Proctor and Lu [10], found that the reduction in the magnitude of the Simon effect that occurs as a function of practice may require that location vary in an irrelevant manner. The Simon effect is also affected by the sequence of congruent and incongruent trial presentations (e.g. sequential effects). In addition, Hommel, Proctor, and Vu [11] found that the Simon effect is present after congruent trials, and is reduced or reversed after incongruent trials. These studies suggest that the Simon effect can become reduced due to training, but the underlying mechanism is not entirely clear.

**Figure 1:**
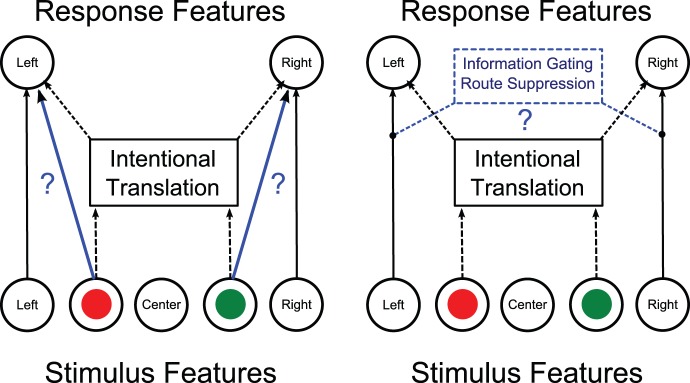
Two competing dual-route models for the Simon task. The broken lines represent intentional processes, and straight lines for automatic processes. The red lines and question marks are the proposed mechanisms for the reduction of the Simon Effect. A. Notebaert et al. [12], attributes the reduction in the Simon effect to associative learning from the specific stimulus-response features. B. Valle-Inclán et al. [13] believes that the reduction of the Simon effect is due to Information Gating/Route Suppression of the automatic processes. Modified from Hommel et al. [11].

There are two models that provide a possible explanation of how the Simon effect is reduced, due to training. The Notebaert et al. [12], model states that the reduction in the Simon effect from training might be due to intentional processes that are strengthened [12]. The Valle-Inclán et al. [13] model states that the reduction of the Simon effect is due to automatic responses that are suppressed or gated [13] (see Figure 1).

The Notebaert et al. [12] model, attributes the reduction in the Simon effect to the interaction between the response color and location repetitions. When the researchers presented stimuli at the same location in close temporal succession, no significant difference in the reaction times between congruent and incongruent trials was found. This suggests that attentional shifts are a necessary and sufficient condition for the Simon effect to occur. The direction of the shift towards the stimulus, rather than the relation of the stimulus to a referent, causes the Simon effect. This would be consistent with the attention-shift hypothesis that argues for a spatial stimulus code that originates in the movement of an attentional spotlight [14]. This model is largely based on a model of trial-by-trial reductions in the Simon effect. However, it is not entirely clear if it also includes general training that might occur over multiple sessions.

Valle-Inclán et al. [13] observed the disappearance of the Simon effect when the participants were presented with incongruent trial conditions. This model suggests that the automatic response may be influenced by voluntary control. Stürmer et al. [15] has hypothesized that control over both routes of information processing in the Simon task is possible.

For investigations that examined perceptual learning, transfer designs are heavily used [16]. Transfer designs can be used to evaluate the nature of the changes in information processing that occurs as a function of training [17]. The specificity of learning can be evaluated by comparing the conditions for which transfer has occurred with those for which it did not [18]. Studies using practice transfer designs [10] have shown that learning can modulate the Simon effect. Different kinds of tasks when performed before the Simon task, eliminate or reverse the Simon effect [19] [20].

To resolve the controversy between the Notebaert et al. [12] and Valle-Inclán et al. [13] models, we examined how training on a Simon task with color would transfer to performance on a Simon task with shape. For the present study, the main hypothesis is that intentional processes are strengthened and/or the automatic responses may be suppressed/gated with training. If the Notebaert et al. [12], model is correct, then the color and the location will impact performance when subjects are asked to respond for shape. For example, if an association between red and right was made, then a reduced reaction time should be observed when the shapes have a consistent color and location (e.g. red and right). These effects should be most noticeable during trials where the stimuli are located in a central position where the confounding of location is not present. These effects should also be observable during lateralized trials, as an additive effect.

If the Valle-Inclán et al. [13] model is correct, then we should observe an overall reduction of the Simon effect during the shape tests, but with no influence on the reaction time for color location associations. The route suppression/gating model would predict that training would carry over to a testing session where the associative learning conditions of color are no longer part of the implicit task. We should also see no significant difference during the testing session with centrally located trials when the shape and color are consistent versus inconsistent with the training.

In a broader sense, our study was aimed at understanding general mechanisms related to cognitive control in sensory-motor tasks. Understanding how conflicting sensory information is resolved through task-experience and the extent to which this knowledge can be transferred to similar tasks has broad applicability to how we are able to perform tasks with “unnatural” stimulus-response mappings. In many modern contexts, such as computer and tool use, driving, sports, dental procedures, we are faced with pre-potent stimulus-response conflicts, in some cases, like laproscopic surgery, these can be life threatening. A better understanding of how humans are able to learn to overcome such conflicts can be of benefit in training people to do better at these tasks.

## Materials and Methods

### Participants

Sixty-three students enrolled in introductory psychology classes at University of California, Riverside, participated to fulfill a course requirement. Thirty-six subjects were run in the main procedure, fourteen subjects were used to assess the continued suppression of the Simon effect and thirteen subjects used to control for training effects in the shape task. Subjects were required to have normal or corrected-to-normal vision in order to participate (self reported). Informed consent was obtained in writing from all the subjects and the experiments were conducted in accordance with the IRB approved by the Human Research Review Board at the University of California Riverside. The subjects were naïve as to the purpose of the experiment.

### Apparatus

A Macintosh G4 (Apple) computer was used to generate the stimuli and record the responses. A custom program was written in Matlab (The MathWorks, Natick, MA) using the Psychophysics Toolbox [21]. The stimuli were displayed on a Mitsubishi Diamond Pro 2070SB monitor with a resolution of 1024×768 pixels using a refresh rate of 100 Hz. The experiments were conducted with binocular viewing during all conditions. Subjects sat on a height adjustable chair, and a chin rest was used to reduce head movements during the task. The subjects responded with an Attack 3 Joystick (Logitech).

### Procedure

Subjects were seated in a dimly lit, sound-attenuated testing room. They were first familiarized with the testing equipment, procedure. They were instructed to maintain their visual focus on a central fixation point displayed on the computer screen. The main experiment consisted of 5 days. At the end of each block, they were given feedback based on the percentage of trials that were correct, and the amount of money that they earned so far. They were paid up to five dollars a day based on the percentage of correct trials, for a total up to 25 dollars.

#### Practice

On the first day, a practice session (24 trials) was used to acquaint subjects with the Simon Task. A practice session was administered for both the Training (Simon task by color) and for the Testing (Simon task by shape). The subjects received feedback from a computer generated percentage score at the end of each block (12 trials). During the practice session, the experimenter remained in the testing room and carefully monitored the subjects to ensure that they were performing the procedure correctly.

#### Simon task by color (Training)

During the first four days, subjects trained on a Simon task by color with 960 trials per day. Within each trial block, the subjects were presented with circles that were randomly displayed an equal number of times between center, left, and right locations on the display screen. Each subject received a unique randomization. The subjects were instructed to move a joystick to the right or to the left when they saw a circle (diameter 0.6 degrees of visual angle) appear on the screen (e.g. red to the right and green to the left). The stimulus features/response locations were counterbalanced for the red/green versus left/right conditions. They were told to ignore the location of the stimulus and to respond as quickly and as accurately to the color. They were prompted to return the joystick to the exact center position at the end of each trial. During each daily session, subjects were given a break for up to 1 minute after every 48 trials.

After the first 14 subjects were run, a mini color test was added on the fifth day to verify the reduction of the Simon effect due to the 4 days of training (48 trials) (n = 22). The mini test was a Simon task based on color, except with a break and feedback every 12 trials. At the end of each block, the subjects were given computer-generated feedback based on the percentage of trials that were correct, but were not paid money for those trials. This was administered prior to the Simon Task based on shape (testing session).

#### Simon task by shape (Testing)

In the shape-based Simon task, subjects were asked to move a joystick to the right or to the left when they saw a shape appear on the screen (e.g. a triangle to the right and a square to the left). They were told to ignore the location and color of the stimulus and base the responses on the task-relevant shape.

A separate, control group, of subjects (n = 14) was given the shape test without the four days of color-training. This was a different group of subjects to the control one used to assess the continued suppression of the Simon effect.

### Analysis

The relative latency in reaction times is the standard measure of the Simon Effect [3], [11], [22], [23]. The Simon effect was calculated by subtracting the congruent mean reaction times from the incongruent mean reaction times for each condition and day respectively. Planned comparisons between sessions were performed by means of two tailed t-tests (Bonferroni corrections). Results were averaged over all subjects and error bars in the figures are standard errors. For the analysis of the Simon effect, we only used trials where the subjects responded correctly. The mean accuracy rate was 98% after training, so very few trials were eliminated from each session and respective condition.

## Results

### Training

**Figure 2:**
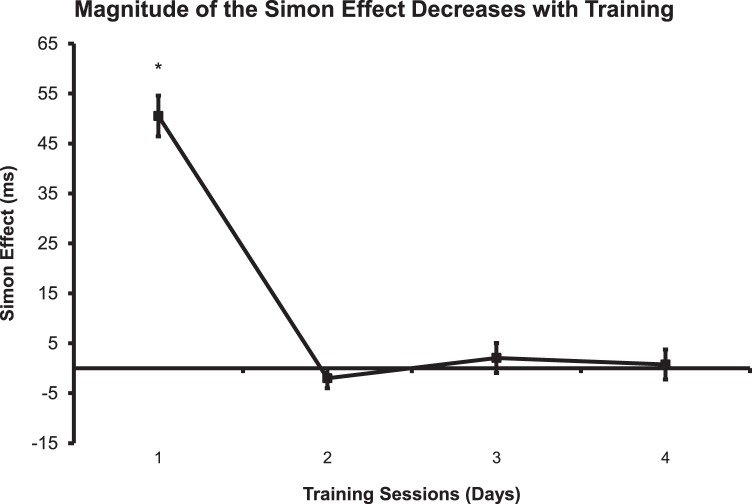
Simon Effect for each session in a choice reaction time color training task. The Simon effect was calculated by subtracting the mean reaction times from the congruent and incongruent conditions for each training session (day). The error bars are Simon effect standard errors. We found that a robust Simon effect persisted only during the first day of training.

During the first day of training, a robust Simon effect was present (50.5 ms ±4.1 SEM). There was a clear effect of training on the Simon effect over the 4 days of training [F(1,21) = 58.313, p<0.001, one-way repeated-measures ANOVA]. The Simon effect was reduced to the point of near extinction by the second day of training and remained suppressed for days 3–4 (see Figure 2). The mean reaction times for Congruent vs Incongruent conditions persisted only during the first day of training [t(21) = −12.355, p<.001, Post hoc paired t-tests (Bonferroni corrections).

### Assessment of Continued Suppression

**Figure 3:**
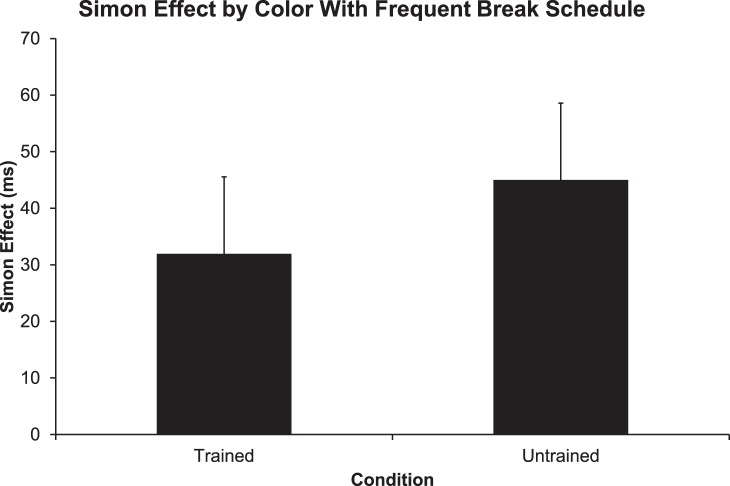
An unexpected return of the Simon effect occurred with a short break schedule. However, the effect of trained versus untrained conditions was not significant.

On the fifth day we administered a mini color based Simon task consisting of only 48 trials, with a break every 12 trials (n = 22) (Figure 3). We wanted to determine if the Simon effect was still suppressed on the fifth day, prior to testing. An unexpected return of the Simon effect (31.9 ms ±5.3 SEM) was found with the trained group using a break schedule that occurred after every 12 trials [t(21) = −6.074, p<.001,paired t-test]. As an additional control, we tested a new group of subjects that did not receive any training (n = 13). The Simon effect (45 ms ±7 SEM) was slightly larger for the untrained control but, the two groups were not statistically different from one another [t(33) = −1.496, p>.01, independent t-test].

**Figure 4:**
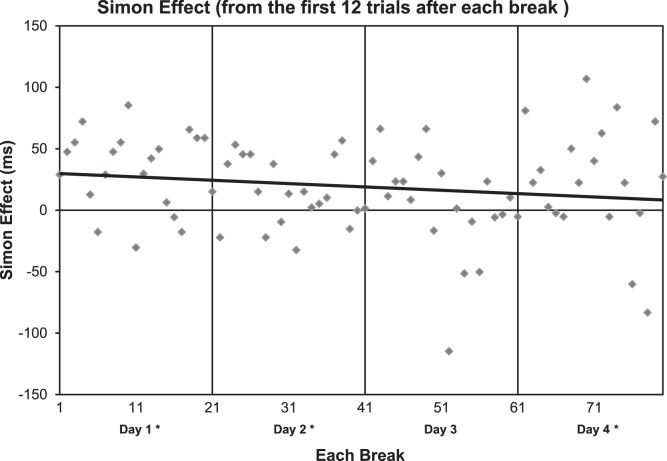
Simon effect calculated from first 12 trials after each break from the same data as shown in Figure 2. All four days of testing were plotted together for comparison. The Simon effect was present for all days except for day 3. As indicated by the line of best fit, the overall trend of the Simon effect for the 12 trials decreased over the four days of training.

Since the Simon effect was observed with the 12 trials per block in the mini color task (see Figure 3), we wanted to determine if the Simon effect was also present during the first 12 trials after each break during the four primary training sessions (Figure 2). To do this, we analyzed the first 12 trials after each break for the four days of training (One-sample t-tests, Simon effect differences not equal to 0) and plotted them (Figure 4). The Simon effect was present for day 1 (p<0.01), day 2 (p<0.05), day 4 (p<.05), but not day 3 (NS). Figure 4 presents single trial data, with only one data point for each subject so there is a great deal of variance in this plot, however, these results indicate that the suppression of the Simon effect is diminished for the first 12 trials after each break. As indicated by the line of best fit, the overall trend of the Simon effect for the 12 trials decreased over the four days of training (Figure 4). An ANOVA on this data showed a trend of an effect of day [F(3,79) = 2.01, p = 0.1)] and there may be some contribution of outlying points in determining this trend. These results suggest that the Simon effect recovers to some extent after breaks and that much of the learning seems to be related to subjects becoming more adept at suppressing the effect after the first few trials of each block.

### Testing

**Figure 5:**
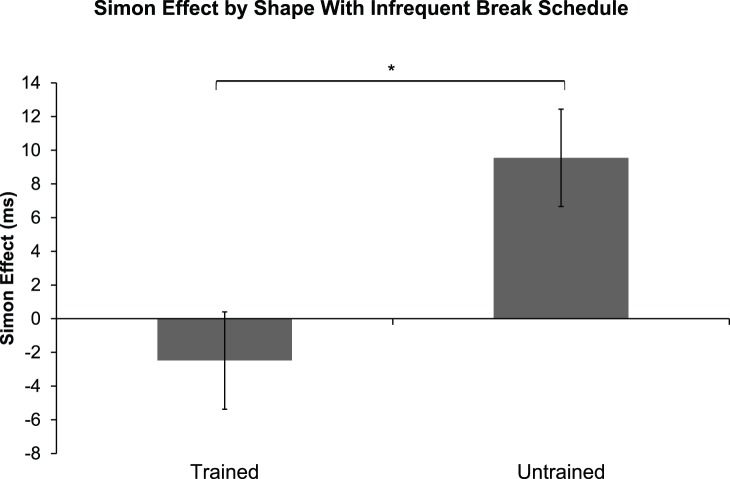
We compared a group that received four days of previous training on a color Simon task, to an untrained group. We found that the effect of training from the previous color Simon task sessions (Figure 1) will reduce the Simon effect for a subsequent shape Simon task.

On the fifth day, an additional testing session (960 trials) was conducted where the task changed, and the subjects had to respond by shape (e.g. triangle to the right and rectangle to the left). They were instructed to ignore the color and location, and respond based solely on the task relevant shape. In figure 5, we show the Simon effect for shapes for the trained (−2.5 ms ±2.9 SEM) versus an additional untrained, control-group (9.5 ms ±4.5 SEM). This difference in the magnitude of the Simon effect for shapes is significantly different between the trained and untrained subjects [f(1) = 4.678, p<.05, one-way ANOVA] and shows that the training based on color transferred as a reduction of effect for the Simon shape task.

### Task-defined Associations

Task-defined associations between response direction and color, Consistent (352.5 ms ±7.33 SEM) versus Inconsistent (353.5 ms±8.09 SEM) groups, did not significantly affect the Simon effect based on shape for lateralized stimuli [f(1) = .034, p>.05, one way repeated measures ANOVA]. For the central stimuli, Consistent (335 ms±9.1 SEM) versus Inconsistent (332 ms±7.3 SEM); no significant associative learning from the specific stimulus-response features was found [f(1) = .362, p>.05, a one way repeated measures ANOVA]. Together, the task defined association tests failed to support the conclusion that the reduction of the Simon effect is due to an association with stimulus features and responses.

## Discussion

The five major points illustrated by the present study, is that (1) the magnitude of the Simon effect decreases with training, (2) the Simon Effect returns in the first few trials after a break, (3) task-defined associations between response direction and color did not significantly impact the Simon effect based on shape, (4) the reduction of the Simon Effect from the training (based on color) transferred to yield a significant reduction of the overall Simon effect based on shape, relative to subjects that did not receive training and, (5) no significant associative learning from the specific stimulus-response features was found for the centrally located stimuli. Together, these results show that the Simon Effect can be ameliorated by training, however, that this learned reduction in the Simon Effect may require online inhibition and that this inhibition transfers in part to other features.

Notably, these results are the first demonstration that the learning effects from a continuous task has a short term rebound effects after a break. After each break the Simon effect returns and once the task becomes repetitive, past around 15 trials, the learned suppression of the Simon effect resumes. We suggest that this short-term rebound effect may be due to an online inhibition process that must be reactivated after each break. This would be consistent with an actively maintained, rather than an automatic, inhibition process.

We failed to find any evidence from the testing sessions that the reduction of the Simon effect was due to an association with stimulus features and responses. As such, our results don't appear to support the Notebaert et al. [12] model. However, if associations between color (stimulus) and location (response) did not transfer over from the Simon color to the Simon shape task, it is still plausible that association learning may still be responsible for some of the effects seen during the shape task. The Notebaert et al. [12] model, attributes the reduction in the Simon effect to associative learning from the specific stimulus-response features (see Figure 1a). No significant associative learning from the specific stimulus-response features was found, for the centralized stimuli. The central (neutral) stimulus condition was used to determine if we could observe reaction time differences without confounding stimulus location. Together our results fail to support the Notebaert et al. [12] model, however since this lack of support is based upon a null result this does not indicate a falsification of their model.

We did find evidence that the training with color improved performance relative to subjects that did not receive training (see Figure 5). This carry over from one task to another is consistent with the findings by Valle-Inclán et al. [13], that attribute the disappearance of the Simon effect following incongruent trials. Our results support this finding by demonstrating that some carry over effects exist.

On the other hand, Hommel et al. [11] has argued that gating/suppression of the automatic response-selection route is not the only candidate explanation for the sequential variation in the Simon effect. Hommel et al. [11] used two stimulus response pairs that were presented during each trial; the first was a prime and the second a probe. In one condition, the participants did not perform the response. The overall finding was that the Simon effect was eliminated when the preceding responses did not depend on the preceding stimulus, or when the preceding trial did not require an actual response. Since an actual response for the previous stimulus was not necessary, the conclusion was that even if gating/suppression is responsible for the sequential effects, it is not under voluntary control.

We also found an unexpected effect from the different break schedules that were used. This suggests that the learned suppression of the Simon effect will degrade over time. This is an intriguing characteristic of the system, and should be investigated more systematically in a future study.

Several investigators have tried to determine the processing stage in which the Simon effect occurs. The Simon effect is considered by some investigators to be a response-selection phenomenon [17]. In essence, the latency is thought to occur at a response-selection stage of information processing, [24], and has been attributed to the suppression of an automatic response-activation route [25].

Other investigators attribute the Simon effect to a type of response competition [26]. The basic argument is that some kind of response code is generated for the stimulus features. These response codes are then used for generating a response. According to Umiltà and Nicoletti [26], a response code is formed relative to the egocentric axes, and another is formed relative to an external reference location. For example, during trials where the irrelevant response code corresponds with the response code signaled by the relevant stimulus dimension, there is no competition. Contrariwise, for trials where the irrelevant response code does not correspond with the relevant response code, it produces competition. This competition of response codes must be resolved before the correct response can be made. It is this response competition that is assumed to be the primary cause of the slower reaction times for the noncorresponding trials relative to the corresponding trials.

Additional alternatives to the response-selection phenomenon have been proposed. Hasbroucq and Guiard, [27], argue that the effect is due to a stimulus-identification process. The Simon effect is a function of stimulus-stimulus congruity, the correspondence between the two dimensions of the stimulus. The assignment of the stimulus property signifies that position. The stimulus event amounts to the presentation of two simultaneous left-right messages. This makes the stimuli either intrinsically congruent or incongruent [27]. Therefore, the stimulus identification process is longer when the identification is prolonged when the irrelevant location of a stimulus (position) is incongruent with the relevant feature dimension.

The explanation by Hasbroucq and Guiard [27], seems to be in conflict with data collected from the Hedge and Marsh task [4]. In brief, the subjects in the Hedge and Marsh task [4] were asked to respond with either the key of the same color as the stimulus or the key of the alternative color. Hedge and Marsh [4] argued that the instructions for this specific task do not directly link stimulus color with response location [28]. Guiard et al. [27] responded by saying that neither version of the Simon task [28], allows one to disentangle irrelevant spatial Stimulus response correspondence and Stimulus congruity.

In a more recent study, a more permanent reversal of the Simon effect was found after incongruent trials, showing that sequential modulations depend on long-term practice effects [29]. However, even short-term associations between stimuli and responses can produce significant effects [30]. Practice and correspondence sequence effects were also found to co-occur and be additive [31]. In the current study, by using a training and testing paradigm we are able to disentangle many of these previous issues. Furthermore, our participants used a joystick with the same arm to respond to all conditions eliminating the possibility of confounds associated from responding with different arms or fingers.

### Conclusion

The main result from the current study is that the magnitude of the Simon effect decreases with training, and is nearly abolished with an infrequent break schedule. The most parsimonious explanation is that subjects learn to more efficiently apply inhibition when appropriate. Notably this learned inhibition can be generalized to other contexts, suggesting that subjects may be more generally learning how to suppress irrelevant information. However, the Simon effect returns in the first few trials after a break suggesting that this may not be an automatic inhibition but requires active maintenance. These results suggest that the underlying conflict that gives rise to the Simon effect is difficult to eliminate with training but that it can be kept in check by learning cognitive control. It is likely that inhibition of prepotent responses in naturalistic contexts suffers from similar constraints.
